# Encephalomyocarditis Virus Entry Unveiled

**DOI:** 10.1128/mBio.00305-19

**Published:** 2019-03-26

**Authors:** Sara Cherry

**Affiliations:** a Department of Microbiology, University of Pennsylvania, Philadelphia, Pennsylvania, USA

**Keywords:** entry, receptor, virus

## Abstract

Picornaviruses are a widespread group of pathogens that can cause diverse pathologies. Pathogenesis is thought to be driven by the tissue-specific tropisms displayed by these viruses. For example, many picornaviruses can infect the heart and cause viral myocarditis. Encephalomyocarditis virus (EMCV) is a rodent pathogen that causes myocarditis in rodent models and has been used to model this biology. However, the receptor and entry requirements for this virus are poorly understood. L. E. Bazzone, M. King, C. R. MacKay, P. P. Kyawe, et al. (mBio 10:e02734-18, 2019, https://doi.org/10.1128/mBio.02734-18) tackled this problem using CRISPR knockout screening in human cells that are susceptible to EMCV and identified ADAM9 as an essential entry factor for EMCV in mouse and human cells. Since the extracellular domain but not the enzymatic activity or intracellular domain is required for infection, the data suggest that ADAM9 acts as an entry receptor or at an early step in the process, shedding light on the biology of EMCV infection and pathogenesis.

## Abstract (precis)

Picornaviruses are a widespread group of pathogens that can cause diverse pathologies. Pathogenesis is thought to be driven by the tissue-specific tropisms displayed by these viruses.

## COMMENTARY

Picornaviruses are a large group of viruses that are emerging and reemerging, and many have narrow host ranges. This is at least in part due to the specificity for the entry receptor, as picornaviruses bind to receptors at the plasma membrane that dictate many aspects of tropism and pathogenesis (1). For example, poliovirus uses poliovirus receptor (PVR) as the entry receptor, and mice transgenic for this human receptor become permissive to infection (1). Therefore, many studies have explored the entry receptors and entry requirements for diverse picornaviruses both to inform the basic biology and to develop new strategies for interventions. Encephalomyocarditis virus (EMCV) exhibits a broader host range than many human picornaviruses, and seroprevalence studies suggest that subclinical infections occur in humans (2). Nevertheless, the entry pathway for EMCV is incompletely understood; we also lack knowledge of the entry receptor. Studies have implicated sialic acid and other factors as attachment factors, but a definitive receptor and many other aspects of the entry pathway remain unclear (2).

To tackle this problem and identify cellular factors required for EMCV infection, Bazzone et al. performed a CRISPR/Cas9 genetic screen (3). Since EMCV is lytic in many cells, including human HeLa cells, they screened for cells that survived two rounds of EMCV infection, identifying nonessential genes either that are required for EMCV replication or that are required for EMCV-dependent cell death. Using this strategy, they identified seven genes, two of which made up more than 90% of the sequenced reads, ADAM9 and PA2G4. Since ADAM9 is a type I plasma membrane protein that is broadly expressed, the authors focused on this candidate as a potential entry factor.

Genetic deletion of ADAM9 in multiple human cell lines and primary mouse lung fibroblasts protected cells from EMCV infection; there was a multilog drop in viral replication and protection from virus-induced cytolysis. Given that different strains of EMCV have differential binding activities to attachment factors, such as sialic acid (2), they tested the role of ADAM9 during infection by a second strain of EMCV. They found that ADAM9 was required for infection by both strains of EMCV but not another picornavirus, Coxsackie virus B3, or unrelated viruses, suggesting specificity.

Next, Bazzone et al. began to dissect the structural features of ADAM9 required for viral infection. ADAM9 is a member of a family of transmembrane metalloproteinases that play important roles in signaling as well as adhesion through proteolytic capabilities that modulate the activities of cytokines, chemokines, growth factors, their associated receptors, and cell adhesion molecules (4–6). Therefore, the authors performed a structure-function analysis. They first verified that wild-type ADAM9 could rescue their knocked-out cells; the complemented cells became permissive to EMCV infection. Second, they tested the role of the extracellular protease in viral infection, and they found that a point mutation that inactivates the protease function remained permissive. This suggests that protease activity is dispensable for infection and that ADAM9 did not indirectly control infection through the cleavage of another host factor. Third, they tested the role of the cytoplasmic tail required for signaling through an SH3 domain and found that this too was dispensable. This suggests that ADAM9 is not required for its signaling capabilities in infected cells. All together, this suggests a more structural role in binding or entry. ADAM9 has a number of extracellular domains, including a disintegrin domain required for interactions with integrins, and the catalytic activity of ADAM9 is independent of interactions with integrins (5). Future studies will define the extracellular domains or regions required for infection.

To begin to address whether entry was indeed the step in infection dependent on ADAM9, Bazzone et al. tested whether they could bypass the requirement for ADAM9 by transfecting viral genomic RNA directly into the cytoplasm of ADAM9 knockout cells. Transfection of viral RNA into the cytoplasm allows the virus to begin replicating, making cellular factors required for entry and uncoating dispensable. Using this strategy, they found that both wild-type and ADAM9-deficient cells could support high levels of infection (3). This shows that ADAM9 is required only during the early steps in the replication cycle. All together, these data support the hypothesis that ADAM9 is an entry receptor for EMCV. Future studies are required to demonstrate direct interactions between the virus and ADAM9 at the membrane.

Interestingly, ADAM9 has been implicated in a number of diseases. While ADAM9 is broadly expressed, increased expression is associated with a wide variety of human cancers, as well as in Alzheimer's disease (6, 7). Recently, loss-of-function mutations in humans have been associated with cone rod dystrophy (CRD), which leads to visual degeneration (6, 14). Likewise, mutations in canine *Adam9* are responsible for the retinal dystrophy occurring in some Glen of Imaal terriers (8). The mouse knockout is developmentally normal but presents with a degenerative retinal disease (9). Importantly, the authors found that lung fibroblasts from the ADAM9 knockout mice are refractory to EMCV infection (3). Therefore, future studies will reveal whether the animals are now refractory to infection and uncover the role of ADAM9 in EMCV pathogenesis, including myocarditis.

This is an exciting time in picornavirus biology, when the use of cell-based mammalian genetics has led to the discovery of a number of receptors and entry factors. While early studies used biochemical strategies to identify picornavirus receptors, many viral receptors were refractory to identification using these approaches (1). The recent renaissance in human functional genomics has reopened the field. Genetic studies using diverse approaches, including RNA interference (RNAi), haploid genetics, and CRISPR have identified an ever-increasing list of receptors and entry factors (for example, see references [Bibr B10][Bibr B11][Bibr B12] and 13). These new findings will allow for the development of new models to study pathogenesis and will ultimately lead to new therapeutics.
